# Pattern of local adaptation to quantitative host resistance in a major pathogen of a perennial crop

**DOI:** 10.1111/eva.12904

**Published:** 2019-12-31

**Authors:** Thomas Dumartinet, Catherine Abadie, François Bonnot, Françoise Carreel, Véronique Roussel, Rémy Habas, Reina Teresa Martinez, Luis Perez‐Vicente, Jean Carlier

**Affiliations:** ^1^ UMR BGPI Univ Montpellier INRA CIRAD Montpellier SupAgro Montpellier France; ^2^ CIRAD UMR BGPI Capesterre‐Belle‐Eau France; ^3^ UMR AGAP Univ Montpellier INRA CIRAD Montpellier SupAgro Montpellier France; ^4^ IDIAF Santo Domingo Dominican Republic; ^5^ INISAV Havana Cuba

**Keywords:** banana, local adaptation, plant pathogenic fungus, plant quantitative resistance, population genetics, *Pseudocercospora fijiensis*, quantitative trait of pathogenicity

## Abstract

Understanding the mechanisms involved in pathogen adaptation to quantitative resistance in plants has a key role to play in establishing durable strategies for resistance deployment, especially in perennial crops. The erosion of quantitative resistance has been recently suspected in Cuba and the Dominican Republic for a major fungal pathogen of such a crop: *Pseudocercospora fijiensis*, causing black leaf streak disease on banana. This study set out to test whether such erosion has resulted from an adaptation of *P. fijiensis* populations, and to determine whether or not the adaptation is local. Almost 600 *P. fijiensis* isolates from Cuba and the Dominican Republic were sampled using a paired‐population sampling design on resistant and susceptible banana varieties. A low genetic structure of the *P. fijiensis* populations was detected in each country using 16 microsatellite markers. Cross‐inoculation experiments using isolates from susceptible and resistant cultivars were carried out, measuring a quantitative trait (the diseased leaf area) related to pathogen fitness on three varieties. A further analysis based on those data suggested the existence of a local pattern of adaptation to resistant cultivars in both of the study countries, due to the existence of specific (or genotype by genotype) host–pathogen interactions. However, neither cost nor benefit effects for adapted populations were found on the widely used “Cavendish” banana group. These results highlight the need to study specific host–pathogen interactions and pathogen adaptation on a wide range of quantitative resistance phenotypes in banana, in order to develop durable strategies for resistance deployment.

## 1 INTRODUCTION

Interest in plant genetic resistance to pathogens for crop disease management has grown in recent years with a view to limiting pesticide use (Pilet‐Nayel et al., 2017). However, pathogen populations are frequently found to adapt, often rendering plant resistances ineffective (Mundt, 2014). Understanding the mechanisms underlying such adaptation, in order to design durable strategies for plant resistance management, means applying concepts from evolutionary biology related to natural selection.

Two categories of plant resistance have been described in the literature (Parlevliet, 2002; Poland, Balint‐Kurti, Wisser, Pratt, & Nelson, 2009): qualitative resistance interacting with the qualitative component of pathogenicity (i.e., the ability of a pathogen to infect a host) and quantitative resistance interacting with the quantitative component of pathogenicity (often called aggressiveness in the plant pathology literature). In the latter case, infection is possible and the level of disease that can be measured on plants infected by fungi depends on the values taken by the quantitative traits involved in the interaction, related to fitness (including infection efficiency, latent period, spore production rate, infectious period and lesion size; Lannou, 2012). Pathogens are able to adapt rapidly to their hosts, resulting from a fast breakdown of qualitative resistances (McDonald & Linde, 2002). Varieties therefore have to be replaced frequently to control pathogens with new resistance genes, and durable strategies have to be defined for resistance deployment (Mundt, 2014). This is all the more important for perennial crops, which are more prone to inoculum build‐up and epidemic disease development, and variety turnover is much slower (Ploetz, 2007). Quantitative resistance may be more suited to such crops as it is generally more durable (Mundt, 2014). This increased durability could be due to a low probability of accumulating all the mutations needed to overcome multiple QTLs (quantitative trait loci) that might be involved in resistance, and/or a combination of different resistance mechanisms difficult to overcome, or acting on different stages of the pathogen life cycle, and/or lower selection pressure exerted on the pathogen population in comparison with qualitative resistance (Pilet‐Nayel et al., 2017). However, erosion of quantitative resistance resulting from an adaptation of pathogen populations has been observed in some annual crop pathosystems (Abang et al., 2006; Andrivon et al., 2007; Cowger & Mundt, 2002; Frézal, Jacqua, & Neema, 2018; Gent, Massie, Twomey, & Wolfenbarger, 2017), with fewer examples for perennial crops (Caffier et al., 2014; Delmas et al., 2016). Thus, quantitative resistance management strategies may also need to be defined according to how pathogens adapt to this type of resistance.

Agricultural landscapes can be considered as a spatially heterogeneous environment for pathogens when varieties are cultivated in different fields as mosaics. These varieties can differ in their level of quantitative resistance to pathogens. Different adaptive patterns can be involved in quantitative resistance erosion in such a context following the general theory of evolution. Divergent selection between different habitats, in the presence of genotype × environment interactions, combined with restricted gene flow, can lead to the so‐called pattern of local adaptation (Kawecki & Ebert, 2004). In such a pattern, resident genotypes in each population have, on average, greater fitness in their local habitat than genotypes evolving in other habitats. Plant diseases result from interactions between the environment, plants and pathogens (the disease triangle framework; Scholthof, 2007). However, in studies on the host adaptation of pathogens, the local host of origin is assumed to be the main habitat, and the local adaptation term refers here to adaptation to a local host (Croll & McDonald, 2017; Kaltz & Shykoff, 1998; Kawecki & Ebert, 2004; Kraemer & Boynton, 2017). In this context, genotype × environment interactions correspond to specific host–pathogen interactions (equivalent to genotype × genotype interactions between pathogens and varieties, Lambrechts, Fellous, & Koella, 2006). Thus, in a plant pathogen, divergent selection between hosts in a heterogeneous agricultural landscape in the presence of specific host interactions and restricted gene flow can lead to a pattern of local adaptation. The existence of specific host–pathogen interactions could be used to design an “evolution‐proof” mixture of varieties (Gallet et al., 2014) or, more generally, durable strategies for resistance deployment in space and time. By contrast, in the absence of such interactions, directional selection on pathogen populations may increase the level of quantitative pathogenicity on all the hosts, as suggested by a modelling approach (Gandon & Michalakis, 2000). The resulting general adaptation could lead to an impasse in the use of quantitative resistance, since greater pathogen aggressiveness may be selected (Zhan, Thrall, Papaïx, Xie, & Burdon, 2015).

Both patterns (general vs. local adaptation) have been described for plant pathogenic fungi in agricultural systems, but published data in this research area are still sparse today and some experimental designs may not have enough statistical power to detect local adaptation (Abang et al., 2006; Andrivon et al., 2007; Caffier et al., 2014, 2016; Cowger & Mundt, 2002; Delmas et al., 2016; Frézal et al., 2018; Gent et al., 2017). Thus, when erosion of quantitative resistance resulting from an adaptation in pathogen populations is observed, it is essential to determine whether or not that adaptation is local and involves specific host–pathogen interactions, using a dedicated experimental design. Furthermore, detecting patterns of local adaptation may provide important insights into the relative strengths of gene flow and host selection in agricultural landscapes (Blanquart, Gandon, & Nuismer, 2012).

Reciprocal transplant experiments are a classical approach to testing for local adaptation by measuring the fitness of populations in their own habitat (sympatry) and when transplanted in other environments (allopatry; Kawecki & Ebert, 2004). However, such an approach is not applicable for a plant pathogen. As an alternative, quantitative traits related to fitness can be estimated under controlled conditions recreating different combinations of plant varieties and pathogen populations (cross‐inoculation experiments), an approach referred to as the “common garden” approach in the evolutionary biology literature (Kawecki & Ebert, 2004). The challenge in common garden studies is to detect when phenotypic differences between populations arise for reasons other than divergent selection (e.g., genetic drift). Sampling designs including replicates, such as paired‐population designs involving different hosts and geographical locations, may help to detect adaptation in such complex situations (Kawecki & Ebert, 2004).

A practical guide to test for local adaptation, comparing different criteria, has recently been published (Blanquart, Kaltz, Nuismer, & Gandon, 2013). The “sympatric versus allopatric” contrast tested using a linear model on a data set taken as a whole is the most powerful test. In cross‐inoculation experiments with plant pathogens, a significant contrast will be expected when the fitness of the pathogen population is greater if inoculated on their host of origin (in sympatry) than on other hosts (in allopatry). Local adaptation can also be investigated by measuring two other contrasts: (a) the “Home versus Away” contrast (ΔHA), which consists in calculating the mean trait value of a population in its “Home” habitat (cultivars of origin here) minus the mean trait value of that population in all the “Away” habitats (cultivars other than the one of origin here), and (b) the “Local versus Foreign” (ΔLF) contrast, which consists in calculating the mean trait value of a “Local” population in its own habitat (cultivars of origin here) minus the mean trait value of all the “Foreign” populations in the same habitat. For host–pathogen interactions, both contrasts may be needed to provide evidence of local adaptation. The share of local adaptation due to the habitat effect is investigated by measuring ΔHA, while the share of local adaptation due to the pathogen is investigated with ΔLF. When population genetics data are available, another method for separating the effect of diversifying selection from neutral genetic drift between populations can be used, by comparing phenotypic differentiation (measured with the *Q*_ST_ parameter from quantitative traits) with genetic differentiation (measured with the *F*_ST_ parameter from molecular neutral markers; Garbelotto, Rocca, Osmundson, di Lonardo, & Danti, 2015; Herrmann et al., 2018; Leinonen, McCairns, O'Hara, & Merilä, 2013). When *Q*_ST_ falls outside the distribution of *F*_ST_, this can be used as an evidence of divergent (*Q*_ST_ > *F*_ST_) or uniform (*Q*_ST_ < *F*_ST_) selection among populations. In the case of plant pathogens, the *Q*_ST_ parameter can be estimated from quantitative traits involved in the interaction with the host, and evidence of divergent selection in such a situation may reflect local adaptation.

Putative erosion of quantitative resistance has recently been observed in a major plant pathogen of a perennial crop: the ascomycete fungus *Pseudocercospora fijiensis* (causing black leaf streak disease [BLSD] on banana). The BLSD pandemic is recent and started around 1960 (see Guzman et al., 2019 for a recent review on BLSD). BLSD is considered to be the most damageable disease of banana worldwide, and several breeding programmes are seeking to create new resistant varieties. Such varieties created by the Fundación Hondureña de Investigación Agrícola (FHIA) were cultivated on a large scale in the 1990s and 2000s in Cuba and the Dominican Republic, respectively. From that time on, agricultural landscapes in some areas became a mosaic of plantations formed by either resistant or susceptible varieties. Signs of quantitative resistance erosion in those varieties were suspected after more than 5 years of cultivation (Guzman et al., 2019; Pérez Miranda, Pérez Vicente, Trujillo, & Betancourt, 2006). A study of the population history of *P. fijiensis* on a global scale showed that genetic diversity and recombination through sexual reproduction exist in all populations around the world (Robert, Ravigne, Zapater, Abadie, & Carlier, 2012). Thus, this fungus may have high evolutionary potential (McDonald & Linde, 2002) and adaptation to host resistances has been suspected.

This study set out first to test whether the erosion of quantitative resistance to *P. fijiensis* in banana varieties has resulted from an adaptation of the pathogen population and, secondly, to determine whether that adaptation is local. To achieve these objectives, *P. fijiensis* isolates were sampled using a paired‐population design on resistant and susceptible banana cultivars in different locations in Cuba and the Dominican Republic. Population structure was first analysed using SSR markers with samples from Honduras as the outgroup (country where BLSD was first introduced in the Latin America–Caribbean zone; Robert et al., 2012). Then, common garden experiments were conducted using cross‐inoculation of banana varieties under controlled conditions, to evaluate quantitative traits involved in interaction.

## 2 MATERIALS AND METHODS

### 2.1 Sampling

Samples from Cuba and the Dominican Republic (DR) were collected in 2011 using a paired‐population sampling design (Table 1). Three different locations 50–300 km apart were sampled in each country, and infected banana leaves were collected. In each location, two banana plantations 2–8 km apart were sampled, one cultivated with a susceptible banana variety and another cultivated with a resistant variety. Given that *P. fijiensis* ascospores can spread for a few 100 m or so (Rieux, Bellaire, Zapater, Ravigne, & Carlier, 2014), distances ranging from 2–8 km were chosen to limit gene flow between plots, which could have counteracted host selection (Lenormand, 2002). The varieties collected were the same within the countries, but they were different between the two countries. The two susceptible varieties sampled were called “Macho3/4” for Cuba and “Macho por Hembra” (abbreviated “Macho” hereafter) for DR. These varieties belong to the genomic group AAB and the plantain subgroup, which is genetically very homogeneous (Hippolyte et al., 2012). The two resistant cultivars called “FHIA18” and “FHIA21” from Cuba and DR, respectively, are both tetraploid hybrids (AAAB group) created by the Fundación Hondureña de Investigación Agrícola (FHIA) with a diploid hybrid (called SH‐3142) resistant to BLSD as a common male parent and different triploids susceptible to BLSD as the female parent (Barekye, 2011; Irish, Goenaga, Rios, Chavarria‐Carvajal, & Ploetz, 2013). Samples from a location in Honduras, the country where BLSD was first introduced in the Latin America–Caribbean zone (Robert et al., 2012), and two plots containing two different susceptible varieties (“French sombre” another plantain and “Grande naine” belonging to the AAA group and Cavendish subgroup) were included as reference populations. Around 50 necrotic leaf fragments were collected per plot (1 fragment/banana) and placed over a culture medium allowing ascospore discharge according to the protocol of Zapater, Abadie, Pignolet, Carlier, and Mourichon (2008). One isolate was isolated per banana. Mycelium cultures from single ascospores were identified as belonging to the species *P. fijiensis* and stored as described in Zapater et al. (2008). In all, 598 *P. fijiensis* strains were isolated.

**Table 1:** Samples of *Pseudocercospora fijiensis* studied

Country	Location name	Population code	Cultivar of origin			Number of isolates	
			Name	Phenotype	Group	Genotyped	Phenotyped
Cuba	Villa Clara	CU1 S2	Macho 3/4	Susceptible	AAB	40	32
		CU1 R2	Fhia18	Resistant	AAAB	48	31
	Ciego de Avila	CU2 S2	Macho 3/4	Susceptible	AAB	52	16
		CU2 R2	Fhia18	Resistant	AAAB	62	16
	Matanzas	CU3 S2	Macho 3/4	Susceptible	AAB	46	16
		CU3 R2	Fhia18	Resistant	AAAB	42	16
Dominican Republic	La vega	DR1 S1	Macho	Susceptible	AAB	42	21
		DR1 R1	Fhia21	Resistant	AAAB	35	21
	Moca	DR2 S1	Macho	Susceptible	AAB	36	0
		DR2 R1	Fhia21	Resistant	AAAB	34	0
	San Francisco	DR3 S1	Macho	Susceptible	AAB	47	23
		DR3 R1	Fhia21	Resistant	AAAB	57	23
Honduras	La Lima	HN1 S3	Grande Naine	Susceptible	AAA	27	0
		HN1 S4	French Sombre	Susceptible	AAB	30	0

Note

Information about the sampling location (country, location), the code associated with the populations (population code) and about the cultivar of origin (name, resistance phenotype and banana genetic group) are presented. The numbers of *Pseudocercospora fijiensis* isolates genotyped using microsatellite markers and phenotyped for quantitative pathogenicity are also given.

### 2.2 Population genetic structure

The population genetic structure was described using the 598 isolates genotyped with 16 microsatellite markers. These markers had already been used in other studies (Neu, Kaemmer, & Kahl, Fischer, & Weising, 1999; Robert, Rieux, Argout, Carlier, & Zapater, 2010; Zapater et al., 2008). They were combined in three multiplex panels of four markers for the first panel and six markers for the other two panels (Table S1). PCR amplification and genotyping were carried out as described in Robert et al. (2010). The population genetic structure was described by measuring several indices using several packages of R 3.6.0 (R Core Team, 2019). Gene diversity (H_E_; Nei, 1978), Simpson's index (*λ*) and the standardized index of association (${\bar{r}}_{d}$) were estimated using the *poppr* R‐package (Kamvar, Tabima, & Grünwald, 2014). Allelic richness (Ar) was estimated using the rarefaction method implemented in the *hierfstat* R‐package (Goudet, 2005). Linkage disequilibrium between each pair of loci in each population was estimated using the *test LD* function implemented in the *genepop* R‐package with default parameters (Rousset, 2008). An AMOVA was carried out using the *varcomp.glob* function implemented in *hierfstat*, in order to estimate molecular variance components on different hierarchical levels: between countries, between locations within countries, between populations within countries and within locations. Lastly, differentiation between countries and between populations was evaluated by calculating pairwise *F*_ST_ (Weir & Cockerham, 1984) using the *pairwise.WCfst()* function implemented in *hierfstat* and the average *F*_ST_ for each country was calculated using the *wc* function of the same package. Genotypic differentiation for all pairs of populations was also tested by performing Fisher's exact tests with the *test_diff()* function implemented in *genepop*. The previously mentioned pairwise *F*_ST_ was then transformed following the formula *T* = −log(1 − *F*_ST_) described by Cavalli‐Sforza (1969) to obtain a matrix of population divergence time. This matrix was used to draw up a neighbour‐joining dendrogram with the *hclust* function implemented in R.

### 2.3 Evaluation of quantitative pathogenicity

As it was not possible to evaluate quantitative pathogenicity (aggressiveness) on all the isolates, a random subsample of 215 isolates (127 isolates from Cuba and 88 from DR, detailed in Table 1) was taken to evaluate quantitative pathogenicity (aggressiveness) through a common garden experiment. The quantitative trait of pathogenicity measured for each isolate was the total diseased leaf area on detached leaf fragments following the protocol described in Abadie, Zapater, Pignolet, Carlier, and Mourichon (2008). Briefly, banana plants multiplied in vitro were cultivated in a greenhouse for 5–7 months under a 12‐hr light photoperiod at 25°C and 85% relative humidity. Leaf fragments (squares of about 36 cm^2^) were collected from the first two deployed leaves of the plants and placed in a Petri dish with the upper leaf surface placed downwards on the survival medium (0.4% bacto agar amended with 5 mg/L of gibberellic acid). Conidial suspensions were prepared from in vitro cultures on a sporulation medium, and desired concentrations (from 10 to 30,000 conidia/ml) were adjusted using a particle counter (Beckman‐Coulter Z1 Coulter Particle Counter). The lower surface of the leaf fragments in the Petri dish was then inoculated with 0.5 ml of conidial suspensions using a microsprayer at constant spraying pressure (about 1.5 kg/cm^2^). After inoculation, the inoculated fragments maintained on survival media were randomly incubated in a climate chamber at 25°C with a 12‐hr light period. When the first symptoms appeared on the inoculated fragments (approximately 30 days postinoculation [dpi]), all fragments were scanned every 10 days, that is at 30, 40, 50 and 60 dpi. All the scans were analysed with an image analysing tool based on the EBImage R‐Package to measure the total diseased area (cm^2^) per leaf fragment.

Cuban and Dominican isolates were inoculated on the resistant cultivars from which they were isolated (“FHIA18” and “FHIA21,” respectively) and on the susceptible cultivars “Macho” and “Cavendish.” As the plantain cultivars “Macho3/4” and “Macho” are very close genetically, only “Macho” was used in these experiments. Cuban and Dominican isolates were inoculated following two different designs. For the 127 Cuban isolates, a conidial suspension at three inoculum concentrations (30,000, 20,000 and 10,000 conidia/ml) was prepared. In order to consider a concentration as a covariable in the linear model used below, each isolate was inoculated on the three cultivars at the three concentrations and the entire experiment was replicated three times (3 × 3 = 9 measurements per isolate–cultivar pair in total). Based on the results from this first design, which revealed highly significant effects for factors of interest, a less time‐consuming design was defined for the Dominican isolates. For these 88 Dominican isolates, a single conidial suspension was prepared at 15,000 conidia/ml from the dilution and the exact final concentration was measured using the particle counter. Each isolate was inoculated on the three cultivars with four replications (4 measurements per isolate–cultivar pair in total).

### 2.4 Statistical analysis

Experiments with the Cuban and Dominican samples were analysed separately at the four data collection times (30, 40, 50 and 60 dpi). The variable was transformed using the square root of the total diseased leaf area in order to obtain the normality of the residuals. The distribution of the residuals was compared to a theoretical normal distribution by plotting normal Q–Q plots. Transformed variables were included in a linear model with mixed effects using the *lmer* function implemented in the *lme4* R‐package (Bates, Mächler, Bolker, & Walker, 2015). The linear mixed‐effects model could be written as follows:

$$Y_{\mathrm{ijklr}}=\mu +f_{r}+\lambda x_{\mathrm{ijkr}}+a_{i}+b_{j}+d_{l}+{\left(\mathrm{ab}\right)}_{\mathrm{ij}}+{\left(\mathrm{ad}\right)}_{\mathrm{il}}+{\left(\mathrm{bd}\right)}_{\mathrm{jl}}+{\left(\mathrm{abd}\right)}_{\mathrm{ijl}}+C_{\mathrm{ijk}}+E_{\mathrm{ijklr}}$$

where *Y_ijklr_* is the variable observed for isolate *k*, sampled on cultivar of origin *j*, in location *i*, and inoculated on test cultivar *l*, in replicate *r*. The covariate *x_ijkr_* corresponds to the inoculum concentration measured using the particle counter for isolate *k*, sampled on cultivar of origin *j* in location *i*, for the experiment replicate *r*, and *λ* is the regression coefficient associated with the concentration. The term *μ* is the intercept, *f_r_* is the effect of replicate *r*, *a_i_* is the effect of location *i*, *b_j_* is the effect of cultivar of origin *j*, *d_l_* is the effect of inoculated cultivar *l*, and *C_ijk_* is the random effect corresponding to isolate *k*. The term (*ab*)*_ij_* is the interaction between location *i* and cultivar of origin *j*, (*ad*)*_il_* is the interaction between location *i* and inoculated cultivar *l*, (*bd*)*_jl_* is the interaction between cultivar of origin *j* and inoculated cultivar *l*, (*abd*)*_ijl_* is the interaction between location *i*, cultivar of origin *j*, and inoculated cultivar *l*, and *E_ijklr_* is the residual error. For the experiment with the Dominican isolates, the “replicate” factor (*f_r_*) and the covariable corresponding to inoculum concentration (*x_ijkr_*) were removed because this experiment was not replicated.

The experimental effects included in the models were investigated by performing a type III analysis of variance (ANOVA) with Satterthwaite's method implemented in the *lmerTest* R‐package (Kuznetsova, Brockhoff, & Christensen, 2017), and least‐square means were computed from the models using the *lsmeans* R‐package (Lenth, 2016). The least‐square means (LSMeans) are the predicted means calculated as the sum of the estimated effects of the model. LSMeans were computed in each country considering different subsets of samples. The *contrast* function of the *lsmeans* package was used to measure the difference between LSMeans of interest. The significance of the contrasts was tested by doing *t* tests and adjusted to the number of tests using the Šidák correction method (Sidak, 1967) implemented in the *lsmeans* package. The measured LSMeans were graphically represented using the *ggplot2* R‐package. As the LSMeans were computed from the square root of the total diseased leaf area, the units on plots are expressed in centimetres.

As we did not sample the “Cavendish” cultivar, the diseased leaf area measured on “Cavendish” was not used to estimate ΔHA and ΔLF contrasts as defined in the introduction (Blanquart et al., 2013). The ΔHA and ΔLF contrasts considering only the resistant and the susceptible cultivars sampled were calculated and tested using the same procedure used above for the other contrasts.

### 2.5 *Q*_ST_‐*F*_ST_ analyses

*Q*_ST_ values were calculated following the formula, $Q_{\text{ST}}=\sigma_{\text{GB}}^{2}/\left(\sigma_{\text{GB}}^{2}+\sigma_{\text{GW}}^{2}\right)$, where $\sigma_{\text{GB}}^{2}$ is the between‐population component of variance and $\sigma_{\text{GW}}^{2}$ is the within‐population component of variance (Spitze, 1993). Variance components were estimated using a linear model for each country inspired by the model published in Lind, Ingvarsson, Johansson, Hall, and Johansson (2011), with the total diseased leaf area measured either on “Macho” or “FHIA” cultivars as the response variable and the “cultivar of origin” as the factor. For the experiment with Cuban isolates, the “inoculum concentration” as the covariable and the “replicate” as the factor were added for a better estimation of variance due to the cultivar of origin. ANOVAs were performed on these models to estimate the between‐population and within‐population components of variance. Then, *Q*_ST_ values within each country were compared to the *F*_ST_ estimated previously within each country using the AMOVA. The difference *Q*_ST_‐*F*_ST_ was calculated and compared to a simulated distribution of *Q*_ST_‐*F*_ST_ values expected under the null hypothesis of evolution in a purely neutral trait, following the procedure described in Lind et al. (2011). The p‐value was calculated as the cumulative probability of the simulated distribution for values greater than or equal to the observed value.

## 3 RESULTS

### 3.1 Low within‐country genetic structure

**Figure 1:**
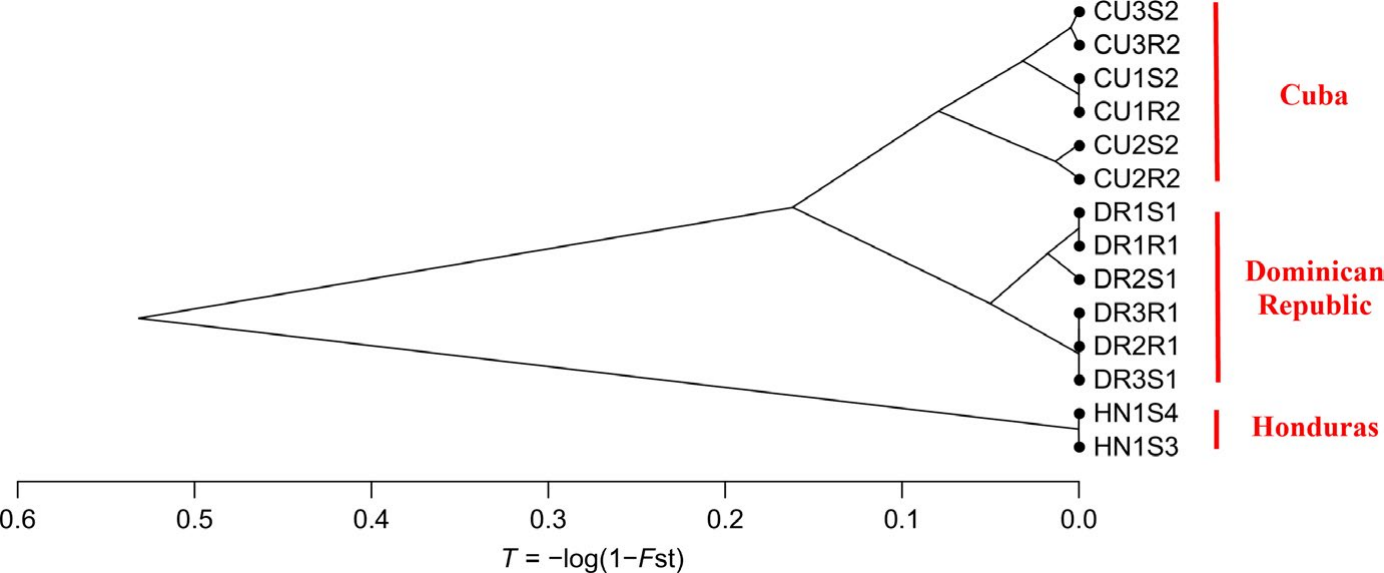
Dendrogram constructed from population divergence time *T* (Cavalli‐Sforza, 1969) calculated between population pairs using 16 microsatellite markers

A high level of genotypic diversity was detected in all the populations with a Simpson's index (*λ*) close to 1. No significant linkage disequilibrium was detected using either the standardized index of association (${\bar{r}}_{d}$) or pairwise Fisher exact tests between loci, showing the occurrence of random mating in all the populations. Genetic diversity in Honduras, the country where the disease was first introduced on that continent, was greater in comparison with the other two countries (Table 2), in accordance with results published by Robert et al. (2012). *F*_ST_ values were calculated between pairs of populations (Table S2) to investigate the structure and relationship between the 14 populations analysed. Pairwise *F*_ST_ values were used to compute the divergence time *T* (Cavalli‐Sforza, 1969) between populations and an unrooted neighbour‐joining dendrogram (Figure 1). The latter provided an overview of the genetic relationship existing between populations from Cuba, the Dominican Republic and Honduras. The Honduran populations were the most differentiated from the Cuban populations, with *F*_ST_ values ranging from 0.25 to 0.39 and from the Dominican populations, with *F*_ST_ values ranging from 0.35 to 0.41 (Table S2). Differentiation between Cuba and the Dominican Republic was somewhat less with *F*_ST_ values ranging from 0.04 to 0.15. The results of the AMOVA (Table 3) revealed that differentiation was relatively high between the 14 populations (*F*_ST_ = 0.20), explaining 79.84% of the total molecular variance. This differentiation was mostly distributed between countries, with a significant *F*_CT_ of 0.18 corresponding to 17.99% of the total molecular variance. The differentiation between populations within countries was low but significant (*F*_SC_ = 0.026, corresponding to 1.88% of the total variance) in Cuba only (*F*_ST_ = 0.04).

**Table 2:** Genetic diversity indices estimated from 16 microsatellite markers in *P. fijiensis* populations and in the studied countries taken as a whole (in bold)

Countries	Population code	Number of isolates	H_E_	Ar
Cuba	CU1 S2	40	0.41	2.08
	CU1 R2	48	0.42	2.16
	CU2 S2	52	0.34	1.84
	CU2 R2	62	0.32	1.74
	CU3 S2	46	0.36	2.01
	CU3 R2	42	0.35	1.96
	**Total**	**290**	**0.37**	**2.32**
Dominican Republic	DR1 S1	42	0.32	1.74
	DR1 R1	35	0.33	1.67
	DR2 S1	36	0.28	1.59
	DR2 R1	34	0.31	1.7
	DR3 S1	47	0.31	1.76
	DR3 R1	57	0.31	1.71
	**Total**	**147**	**0.32**	**1.83**
Honduras	HN1 S3	27	0.51	2.37
	HN1 S4	30	0.5	2.28
	**Total**	**57**	**0.51**	**2.67**

Note

Ar, allelic richness corrected for sample size; H_E_, unbiased estimate of gene diversity (Nei, 1978).

**Table 3:** Analysis of molecular variance (AMOVA) of 14 Caribbean populations of *P. fijiensis* based on 16 microsatellite markers

Data set	Number of populations	Percentage of variation				*F*‐statistics	
		Between countries	Between locations within countries	Between populations within locations	Within locations		
Cuba	6	–	3.36	0.36	96.27	*F*_ST_	**0.037**
DR	6	–	1.26	0.33	98.40	*F*_ST_	0.016
Honduras	2	–	–	1.33	98.67	*F*_ST_	0.000
Global	14	17.99	1.88	0.29	79.84	*F*_SC_	**0.026**
						*F*_CT_	**0.180**
						*F*_ST_	**0.202**

Note

The percentage of variation was estimated on four hierarchical levels: between countries, between locations within countries, between populations within locations and within locations. The AMOVA was performed with the whole data set (global) and for each country. *F*_ST_ measures differences between all populations, *F*_CT_ measures differences between countries and *F*_SC_ measures differences between populations within countries. Significant *F*‐statistics values are indicated in bold (*p*‐value < .05).

### 3.2 Significant factor effects on pathogen populations

An analysis of variance of the total diseased leaf area was carried out for samples from both countries, on each date (30, 40, 50 and 60 dpi). Only the results for 60 dpi (Table 4) are presented, because the effects were more pronounced at the end of the experiments than at early stages of the disease.

**Table 4:** Analysis of variance with the total diseased leaf area 60 days postinoculation as the response variable for the Cuban and Dominican experiments

	Source of variation	Total diseased leaf area					
		Sum Sq	Mean Sq	NumDF	DenDF	Fvalue	Pr(>*F*)
Cuba	Replicate	63.43	32.22	2	2,130.65	254.85	**<2.2E‐16**
	Inoculum concentration	5.57	5.57	1	2,221.93	44.04	**4.03E‐11**
	Location	0.84	0.42	2	121.36	3.32	**0.04**
	Cultivar of origin (CO)	0.09	0.09	1	121.01	0.73	0.4
	Inoculated cultivar (IC)	91.22	45.61	2	2,128.23	360.8	**<2.2E‐16**
	Location: CO	0.09	0.04	2	121.17	0.34	0.71
	Location: IC	0.94	0.23	4	2,128.28	1.85	0.12
	CO: IC	2.77	1.38	2	2,128.08	10.94	**1.89E‐05**
	Location: CO: IC	2.67	0.67	4	2,128.37	5.27	**3.18E‐04**
Dominican Republic	Location	0.45	0.45	1	53.51	1.62	0.21
	Cultivar of origin (CO)	0	0	1	53.51	0	0.99
	Inoculated cultivar (IC)	18.19	9.10	2	439.29	32.93	**4.74E‐14**
	Location: CO	5.2E‐3	5.2E‐3	1	53.51	0.02	0.89
	Location: IC	0.9E‐3	4.5E‐5	2	439.29	0.02	0.98
	CO: IC	5.19	2.60	2	439.29	9.4	**1.01E‐04**
	Location: CO: IC	0.11	0.05	2	439.29	0.2	0.82

Note

This table contains the sum of squares (Sum Sq), the mean square (Mean Sq), the numerator degrees of freedom (NumDF), the denominator degrees of freedom (DenDF), the *F*‐Value (*F*value) and *p*‐value (Pr(>*F*)) corresponding to each factor. The DenDF and Pr(>*F*) were calculated using Satterthwaite's method of approximation. Significant *p*‐values are indicated in bold (*p*‐values < .05).

For the Cuban sample, an analysis of variance after 60 dpi indicated that the effects of the “replicate” and “concentration” factors were highly significant (*p* < .0001). The “location” factor was also slightly significant, suggesting differences between the three locations sampled. A highly significant effect associated with the inoculated cultivar (IC) and its interaction with the cultivar of origin (CO) were also detected. For the Dominican samples, a significant effect only for these two sources of variation was detected with the simpler experimental design used. The LSMean values of diseased leaf area were calculated for both countries, in order to further investigate these two effects.

### 3.3 Quantitative host resistance

**Figure 2:**
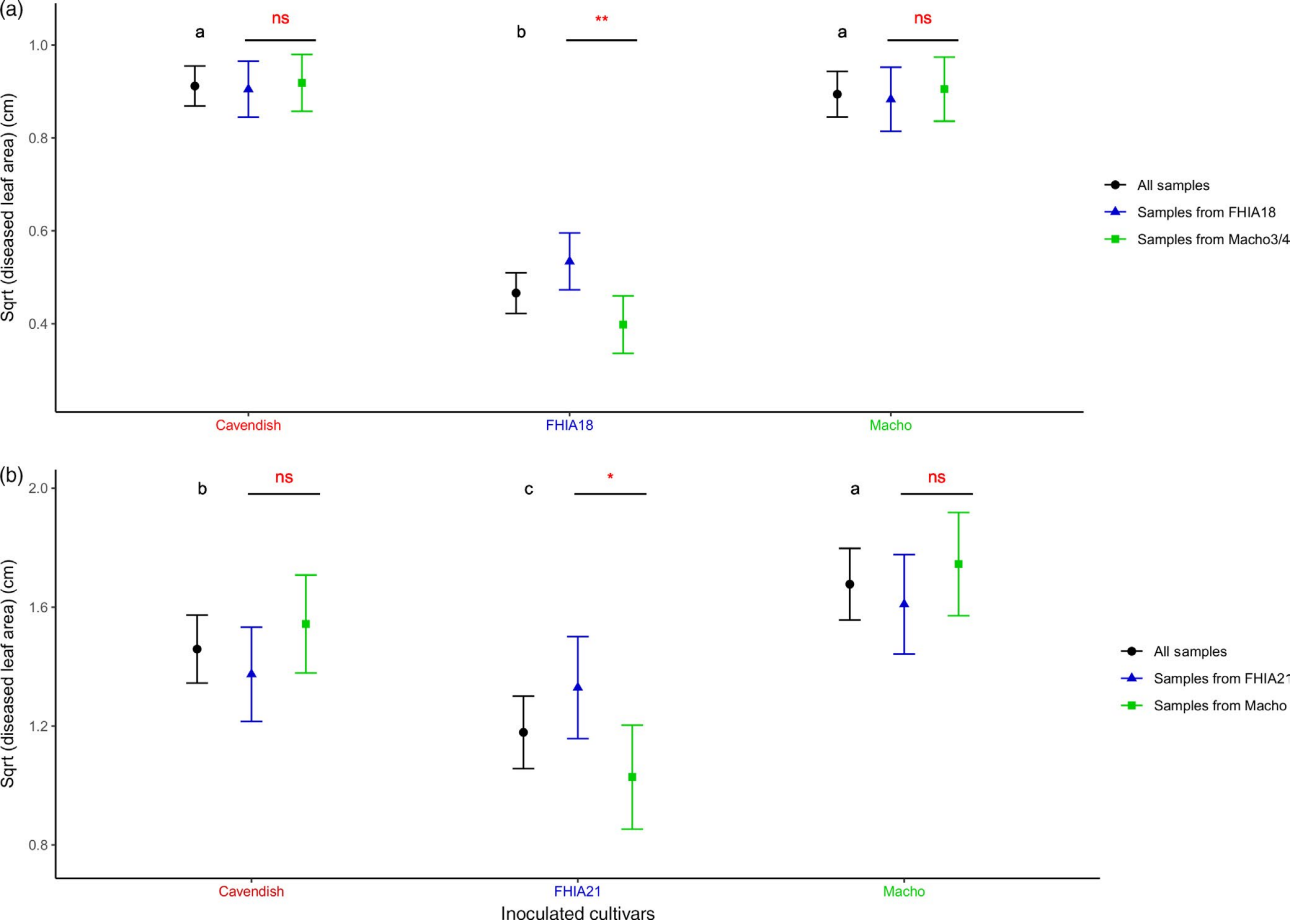
Adjusted means (LSMeans) of the square root (Sqrt) of the total diseased leaf area measured on the different inoculated cultivars considering all the sampled isolates (black), only the isolates sampled on “FHIA” cultivars (blue) or isolated on “Macho” cultivars (green) from Cuba (a) and the Dominican Republic (b). As the LSMeans were computed from square roots, the units are expressed in centimetres. Within each country, LSMeans with the same black letter are not significantly different. Red symbols represent *p*‐values associated with the contrast measured between isolates sampled on FHIA cultivars and isolates sampled on “Macho” (Signif. codes: “***” significant at *p* < .001, “**” significant at *p* < .01, “*” significant at *p* < .05, “n.s” not significant)

In both countries, the effects associated with inoculated cultivars (IC) were significant, suggesting differences between cultivars for the trait measured. LSMeans were calculated for each of the three tested cultivars and on all the isolates from each country (Figure 2, Table S3). The “Cavendish” and “Macho” cultivars had the highest LSMeans compared to “FHIA18” for the Cuban isolates (Figure 2a). The “Macho” cultivar had the highest LSMean, followed by “Cavendish” and then by “FHIA21” for the Dominican isolates (Figure 2b). These results indicate that “FHIA18” and “FHIA21” still expressed some resistance to the studied population in comparison with the susceptible cultivars. No significant difference was observed between “Macho” and “Cavendish” for the Cuban isolates, while “Macho” was significantly more damaged than “Cavendish” for the Dominican isolates.

### 3.4 Local adaptation on resistant cultivars

The ANOVA also indicated significant interaction between the inoculated cultivars (IC) and the cultivar of origin (CO) in both countries. To further investigate this effect, LSMeans were calculated, for each country, for all the (cultivar of origin) × (tested cultivar) pairs (Figure 2, Table S4). For both countries, the differences between the cultivars of origin (resistant vs. susceptible) were only significant for the resistant inoculated cultivars (“FHIA18” for Cuba and “FHIA21” for RD), with the highest LSMeans obtained for the resistant cultivars of origin. The same tendency was observed across locations with LSMeans calculated for all the (cultivar of origin) × (tested cultivar) pairs separately in each sampled location (Figure S1). Significant contrast was only detected for location 1 in Cuba, location where the higher number of isolates was sampled. For Dominican locations, although a highly significant interaction between the inoculated cultivars (IC) and the cultivar of origin (CO) was detected with the ANOVA, the statistical power of independent tests per localities based on small sample sizes was too small to detect significant contrasts. Results were therefore interpreted based only on global analysis, such as the ANOVA.

Based on the LSMeans, the existence of a local adaption pattern in both countries was investigated by measuring and testing Home versus Away (ΔHA) and Local versus Foreign (ΔLF) contrasts (Table 5). For both countries, the two contrasts were estimated from two sets of samples: a first set including all the samples isolated from the susceptible cultivar (“Macho3/4” for Cuba and “Macho” for RD) and a second set including all the samples isolated from the resistant cultivar (“FHIA18” for Cuba and “FHIA21” for RD). The ΔHA values were significantly different from zero regardless of the cultivars of origin. However, in each country, these values were negative for the populations isolated from the resistant cultivars and positive for the populations isolated from the susceptible cultivars. This result suggests that, regardless of the cultivars of origin, all the pathogen populations studied caused more disease on the susceptible cultivars, revealing the difference in cultivar susceptibility to the disease. In contrast, ΔLF was only significantly different from zero for samples isolated from the resistant cultivars of both countries. This observation suggests local adaptation on resistant cultivars.

**Table 5:** Values of local adaptation contrasts based on the HA (Home vs. Away) and LF (Local vs. Foreign) measurements

Country	Cultivar of origin	ΔHA			ΔLF		
		Contrast	*SE*	*p*‐value	Contrast	*SE*	*p*‐value
Cuba	Macho 3/4 (S2)	0.499	0.029	**<.0001**	0.026	0.038	.802
	Fhia18 (R2)	−0.338	0.029	**<.0001**	0.135	0.043	**.001**
Dominican	Macho (S1)	0.725	0.090	**<.0001**	0.147	0.012	.368
Republic	Fhia21 (R1)	−0.294	0.086	**.001**	0.284	0.012	**.035**

Note

These values are provided with standard errors and *p*‐values corresponding to *t* test results done to determine whether ΔHA and ΔLF were significantly different from 0. Statistically significant results (*p*‐value < .05) are indicated in bold.

Local adaptation was also tested using a *Q*_ST_‐*F*_ST_ approach (Table 6). *Q*_ST_ was calculated for the total diseased leaf area between samples from the different cultivars of origin inoculated either on “Macho” (susceptible) or “FHIA” (resistant) in both countries and compared to *F*_ST_ values estimated with the AMOVA. For both countries, *Q*_ST_‐*F*_ST_ calculated on susceptible cultivars was not significantly different from zero. Thus, a selective effect of susceptible cultivars was not detected. In contrast, *Q*_ST_‐*F*_ST_ calculated on resistant cultivars was similar between countries and significantly positive, suggesting divergent selection of these cultivars on pathogen populations.

**Table 6:** *Q*_ST_‐*F*_ST_ analysis between *P. fijiensis* populations collected from susceptible and resistant cultivars in Cuba and the Dominican Republic

Country	*F*_ST_	Inoculated cultivar	*Q*_ST_	*Q*_ST_‐*F*_ST_	*p*‐value
Cuba	0.037	Macho (susceptible)	−1.67E‐03	−0.039	.818
		Fhia18 (resistant)	0.145	**0.108**	**.029**
Dominican Republic	0.016	Macho (susceptible)	0.010	−0.006	.557
		Fhia21 (resistant)	0.095	**0.079**	**.001**

Note

*F*_ST_ values were estimated in both countries using AMOVA and *Q*_ST_ values on the diseased leaf area measured after artificial inoculation on “Macho” cultivars (susceptible) and on “FHIA” cultivars (resistant) in both countries. *Q*_ST_‐*F*_ST_ values significantly different from zero (*p*‐value < .05) are indicated in bold.

There was no significant difference in the measured trait between samples from the different cultivars of origins in each country inoculated on the “Cavendish” cultivars (Figure 2, Table S4). This result suggests that there was no cost or benefit for populations that had adapted to resistant cultivars on this widely used cultivar.

## 4 DISCUSSION

This study set out to determine whether or not the erosion of quantitative resistance in banana cultivars against the fungus *P. fijiensis* has resulted from an adaptation of pathogen populations and what the pattern of such an adaptation might be. Samples were collected using a population‐pair design in different locations in Cuba and the Dominican Republic (DR) on two varieties, one susceptible and one showing quantitative resistance. Phenotypic variation (for a quantitative trait involved in the interaction) using cross‐inoculation experiments and population genetic structure (using microsatellite markers) were evaluated in these samples and a further analysis suggested the existence of a local adaptation pattern on resistant cultivars. In the context of this study, the term local adaptation refers to an adaptation of a pathogen to its local hosts detected from cross‐inoculation experiments (Croll & McDonald, 2017; Kaltz & Shykoff, 1998; Kawecki & Ebert, 2004).

Testing for local adaptation requires estimates of fitness. One common approach is to use one or more individual traits as measures of performance (e.g., infectivity for a parasite), but arguments about their relation to actual fitness have to be provided (Kawecki & Ebert, 2004; Kraemer & Boynton, 2017). A realistic approach in the case of quantitative traits of pathogenicity is to use simulation models to explore their effects on epidemic velocity, which is a good indication of the capacity of the pathogen to invade host populations (Lannou, 2012). A simulation model dedicated to banana BLSD has recently been published (Landry et al., 2017). A sensitivity analysis of the model showed that the three most influential epidemiological parameters are infection efficiency, lesion growth rate and incubation period. The trait measured in our study (the diseased leaf area) actually combined two of the above parameters (lesion growth rate and infection efficiency) and thus appeared to be a good proxy of parasite fitness.

In the evolutionary biology literature, local adaptation is considered as a property of a set of demes within a metapopulation (Kawecki & Ebert, 2004). These authors also consider that the metapopulation is only locally adapted if the fitness of each population at its local site is superior to the average fitness of foreign populations transplanted to that site. The applicability of such a stringent criterion could be limited for agricultural landscapes and the same authors pointed out that it still remains worthwhile identifying subsets of populations showing a pattern of local adaptation and characterizing those subsets by specific properties. Blanquart et al. (2013), proposed a less stringent criterion to test for local adaptation based on a “sympatric versus allopatric” contrast tested using a linear model on a data set taken as a whole. Unfortunately, we could not use this criterion with our design since samples came from only two habitats (two hosts here) and there were not enough degrees of freedom. However, we collected samples using a population‐pair design from different locations and congruent results suggesting local adaptation on resistant cultivars were obtained using complementary analyses on the data set taken as a whole: (a) significant interaction between the cultivars of origin of the populations and cultivars inoculated in a cross‐inoculation experiment was detected measuring the quantitative trait adopted (the diseased leaf area) and using a linear model; (b) using the “local versus foreign” criterion (ΔLF, defined in the introduction) on the same trait, a local adaptation pattern was detected for resistant cultivars in Cuba and the Dominican Republic; and (c) the *Q*_ST_ parameter was estimated from the same trait and a significant *Q*_ST_ > *F*_ST_ was detected on resistant cultivars in both countries, supporting the existence of host selection on those cultivars. Some similar results have been published recently, but only for a few other plant pathogenic fungi (Caffier et al., 2016; Frézal et al., 2018).

Local adaptation was not detected on the susceptible cultivars for which *P. fijiensis* samples were collected when compared with resistant cultivars. The constraints exerted on the pathogen infection of those cultivars might have been lower and did not induce an adaptive response or made it more difficult to detect. A significantly higher level of pathogenicity, regardless the cultivars of origin, was observed for the plantain cultivars (“Macho”) in comparison with “Cavendish,” in the Dominican Republic, but not in Cuba, and a significantly higher level of pathogenicity, regardless the cultivars of origin, was observed for the plantain cultivars (“Macho”) in comparison with “Cavendish,” in the Dominican Republic, but not in Cuba. However, adaptation and host selection between the susceptible cultivars could not be fully tested since isolates from “Cavendish” cultivars were not studied.

The populations locally adapted to quantitative host resistances studied in this paper were not significantly less aggressive on the widely used susceptible cultivars belonging to the Cavendish group. Thus, there was no apparent fitness cost for these populations on the susceptible cultivars as observed for grapevine downy mildew *Plasmopara viticola* (Delmas et al., 2016) and the apple scab pathogen *Venturia inaequalis* (Caffier et al., 2016). However, in contrast with results obtained for the above two pathogens, the *P. fijiensis* populations adapted to resistant cultivars were also not significantly more pathogenic on susceptible cultivars and specific host–pathogen interactions were detected on resistant cultivars. Thus, the emergence of a generalist was not observed. The most important form of genotype × environment interactions for local adaptation is antagonistic pleiotropy, whereby the alleles have opposite effects on fitness in different habitats (Anderson, Lee, Rushworth, Colautti, & Mitchell‐Olds, 2013; Kawecki & Ebert, 2004; Mitchell‐Olds, Willis, & Goldstein, 2007). We could not conclude on the existence of antagonistic pleiotropy in this study, because we did not detect any specific interaction on susceptible cultivars. However, our experimental design was not adapted to detect antagonistic pleiotropy, because lower constraints may have been exerted on the pathogen population by susceptible cultivars. It will be worth testing this hypothesis using more different quantitative‐resistant banana cultivars.

This study provides the first evidence of *P. fijiensis* adaptation to banana quantitative resistance. However, this observation does not necessarily imply that this kind of resistance cannot be used in durable strategies to control BLSD. A pattern of local adaptation to quantitative resistance was detected here, probably resulting from some specific host–pathogen interactions and restricted gene flow between plots only a few kilometres apart. Quantitative resistance has already been described for a wide range of diploid bananas that could be potential parents in breeding programmes (Guzman et al., 2019). If specific host–pathogen interactions exist more generally in these potential parents and involve different alleles or genes among them, trade‐offs in the adaptation of pathogen populations to the different host genotypes might exist. These trade‐offs could then be exploited to define durable disease deployment strategies that constrain pathogen adaptation. The existence of specific host–pathogen interactions in pathogen populations first needs to be studied on a larger number of quantitative‐resistant genotypes. It is also necessary to evaluate how quickly these adaptations take place. Local adaptation frameworks using more quantitative‐resistant genotypes will be useful for conducting such studies. Furthermore, an experimental evolution approach offers great potential for studying the process of local adaptation (Fisher & Lang, 2016; Kawecki & Ebert, 2004; Kawecki et al., 2012). In the case of plant–pathogen interactions such as *P. fijiensis* on banana, experimental evolution in the laboratory is not applicable, but a semi‐experimental approach could be developed based on plots comprising different host‐resistant genotypes under natural infestation and arranged in a way to limit gene flow between them. Following the recent publication of the first reference genome for *P. fijiensis* (Isaza et al., 2016), a population genomics approach could be combined to help understand the process and genetic basis involved in quantitative host adaptation.

## CONFLICT OF INTEREST

None declared.

## Data Availability

All the data (genotypes and phenotypes of *P. fijiensis* isolates) are available in the Data S1.
