# Supplementary material for: Pattern of local adaptation to quantitative host resistance in a major pathogen of a perennial crop
Source: Evol Appl. 2019 Dec 31;13(4):824–36. doi: 10.1111/eva.12904 (PMC7086059; doi:10.1111/eva.12904)
Supplement: Supplementary file 1 [file EVA-13-824-s001.docx]

**Supplementary**

**Table S1:** Multiplexing information and genetic variability of the 16 microsatellite markers used in this study. NA (Number of Alleles) and H_E_ (unbiased estimate of gene diversity (Nei 1978)) for each marker were calculated on the whole sample.

| Multiplex pool | Locus Name | Repeat motif | Reference | Labelling dye | NA | H_E_ |
| --- | --- | --- | --- | --- | --- | --- |
| 1 | MfSSR324 | (TG)_14_ | *Zapater et al. 2008* | NED | 8 | 0.51 |
|  | MfSSR362 | (GA)_4_(…)(GT)_6_ | Zapater et al. 2008 | FAM | 2 | 0.48 |
|  | MfSSR401 | (CTCA)_7_ | *Robert et al. 2010* | PET | 3 | 0.04 |
|  | MfSSR413 | (TCAC)_7_ | *Robert et al. 2010* | FAM | 4 | 0.40 |
| 2 | MfSSR025 | (CA)_18_ | *Neu et al. 1999* | FAM | 5 | 0.29 |
|  | MfSSR406 | (CTTC)_6_ | *Robert et al. 2010* | PET | 2 | 0.46 |
|  | MfSSR407 | (CCAT)_7_ | *Robert et al. 2010* | VIC | 3 | 0.46 |
|  | MfSSR403 | (CAAG)_14_ | *Robert et al. 2010* | NED | 3 | 0.50 |
|  | MfSSR425 | (TGAT)_5_ | *Robert et al. 2010* | PET | 2 | 0.06 |
|  | MfSSR428 | (GAAG)_5_ | *Robert et al. 2010* | NED | 2 | 0.14 |
| 3 | MfSSR061 | (CAA)_8_ | *Neu et al. 1999* | FAM | 4 | 0.14 |
|  | MfSSR381 | (TA)_3_(...)(CA)_3_ | *Zapater et al. 2008* | NED | 2 | 0.43 |
|  | MfSSR402 | (TTGC)_7_ | *Robert et al. 2010* | NED | 2 | 0.42 |
|  | MfSSR412 | (TTGC)_7_ | *Robert et al. 2010* | FAM | 4 | 0.14 |
|  | MfSSR417 | (TTCT) | *Robert et al. 2010* | PET | 10 | 0.54 |
|  | MfSSR424 | (TACC)_5_ | *Robert et al. 2010* | VIC | 3 | 0.17 |

**Table S2:** Pairwise F_ST_ values (Weir and Cockeram) calculated between all population pairs and average F_ST_ values for each country (Bold) and for the global dataset (Red).


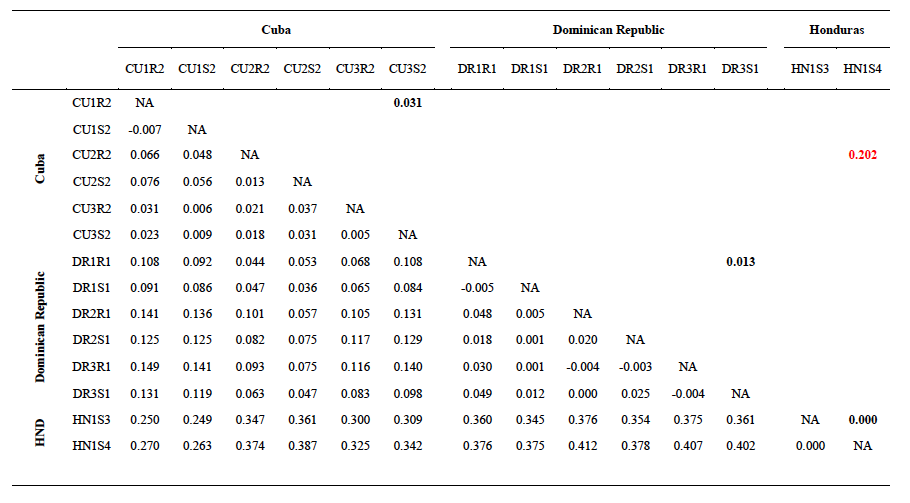


**Table S3:** Adjusted means (LSMeans) and standard error (SE) in cm of the square root (Sqrt) of the diseased leaf area measured in Cuba and the Dominican Republic for each inoculated cultivar. Within each country, LSMeans with the same letter in the "group" column are not significantly different.

| Country | Inoculated cultivars | LSMeans | SE | group |
| --- | --- | --- | --- | --- |
| Cuba | Cavendish | 0.91 | 0.022 | a |
|  | Fhia18 | 0.47 | 0.022 | b |
|  | Macho | 0.90 | 0.025 | a |
| Dominican | Cavendish | 1.46 | 0.058 | a |
| Republic | Fhia21 | 1.18 | 0.061 | b |
|  | Macho | 1.68 | 0.061 | c |

**Table S4:** Adjusted means (LSMeans) and standard error (SE) in cm of the square root (Sqrt) of the diseased leaf area measured in Cuba and the Dominican Republic, for each (cultivar of origin) × (inoculated cultivar) pair. This table also contains the contrast values between the two populations inoculated on a given cultivar and the associated p-value. Statistically significant results (p-value < 0.05) are shown in bold.

| Country | Cultivar of origin | Inoculated cultivars | LSMeans | SE | Contrast | p.value |
| --- | --- | --- | --- | --- | --- | --- |
| Cuba | FHIA18 | Cavendish | 0.905 | 0.031 | -0.014 | 0.985 |
|  | Macho3/4 | Cavendish | 0.918 | 0.031 |  |  |
|  | FHIA18 | FHIA18 | 0.534 | 0.031 | 0.136 | **0.007** |
|  | Macho3/4 | FHIA18 | 0.398 | 0.031 |  |  |
|  | FHIA18 | Macho | 0.883 | 0.035 | -0.022 | 0.958 |
|  | Macho3/4 | Macho | 0.905 | 0.035 |  |  |
| Dominican | FHIA21 | Cavendish | 1.370 | 0.080 | -0.169 | 0.374 |
| Republic | Macho | Cavendish | 1.540 | 0.083 |  |  |
|  | FHIA21 | FHIA21 | 1.330 | 0.086 | 0.301 | **0.048** |
|  | Macho | FHIA21 | 1.030 | 0.088 |  |  |
|  | FHIA21 | Macho | 1.610 | 0.084 | -0.136 | 0.605 |
|  | Macho | Macho | 1.740 | 0.087 |  |  |
|  |  |  |  |  |  |  |

**Figure S1 :** Adjusted means (LSMeans) of the square root (Sqrt) of the diseased leaf area measured in Cuba (A) and the Dominican Republic (B), for each (cultivar of origin) × (inoculated cultivar) pair and each sampled location, considering only the isolates sampled on “FHIA” cultivars (blue) or isolated on “Macho” cultivars (green). As the LSMeans were computed from square roots, the units are expressed in centimetres. Symbols represent p-values associated with the contrast measured between isolates sampled on FHIA cultivars and isolates sampled on “Macho”.

**
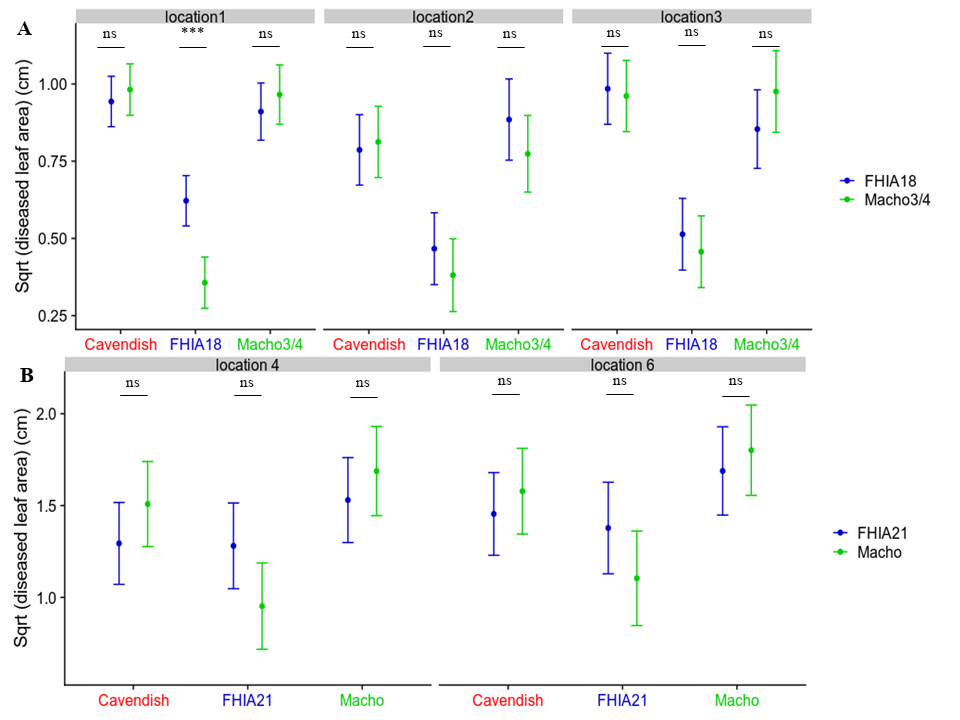
**

(Signif. codes: “***” significant at p < 0.001, “**” significant at p < 0.01, “*” significant at p < 0.05,

“n.s” not significant)
