# Supplementary figures and images for: Pattern of local adaptation to quantitative host resistance in a major pathogen of a perennial crop
Source: Evol Appl. 2019 Dec 31;13(4):824–36. doi: 10.1111/eva.12904 (PMC7086059; doi:10.1111/eva.12904)

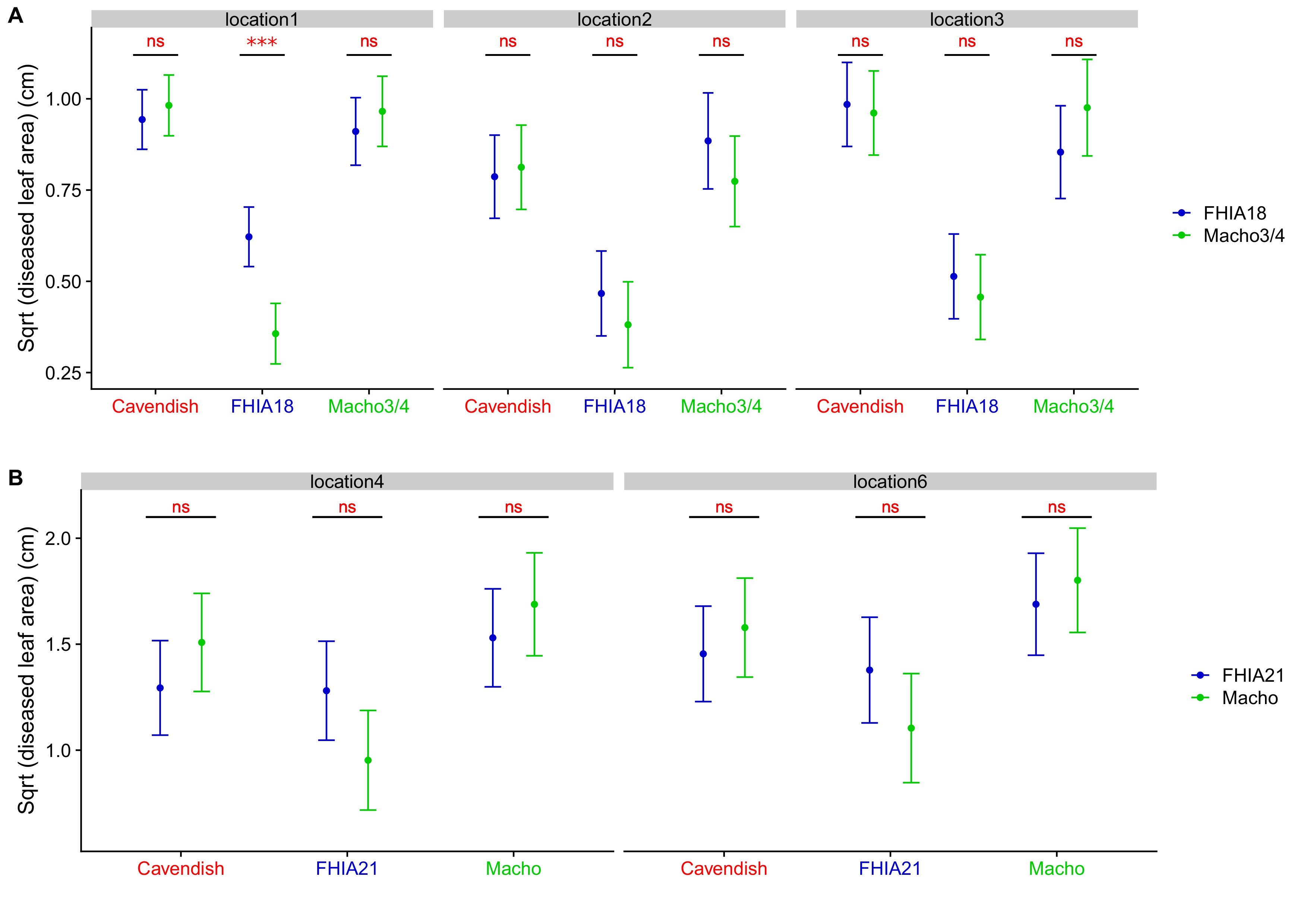

Supplement: Supplementary file 3 [file EVA-13-824-s003.tif]
